# Subtype or state? Rethinking the apCAF paradox and fibroblast plasticity in gastrointestinal cancers

**DOI:** 10.3389/fonc.2026.2000409

**Published:** 2026-10-26

**Authors:** Zhilin Yu, Dingnan Hu, Zhenxiang An

**Affiliations:** 1 Department of Gastroenterology, First Affiliated Hospital of Guizhou University of Traditional Chinese Medicine, Guiyang, China; 2 The First Clinical Medical College, Guizhou University of Traditional Chinese Medicine, Guiyang, China

**Keywords:** antigen-presenting cancer-associated fibroblasts, costimulation, epigenetic reprogramming, fibroblast plasticity, gastrointestinal cancer, MHC class II, tumor microenvironment

## Abstract

Antigen-presenting cancer-associated fibroblasts (apCAFs), commonly defined by MHC class II molecules, CD74, and related transcriptional programs, exhibit divergent functions across gastrointestinal tumors. In pancreatic ductal adenocarcinoma, mesothelium-derived apCAFs promote regulatory T-cell differentiation and suppress effector responses, whereas their abundance in gastric cancer is associated with earlier-stage disease, longer survival, and improved immunotherapy responses. This paradox may reflect microenvironmental context and classifications that conflate transcriptional state, cellular origin, and immune function. apCAFs may therefore represent reversible, context-dependent states defined by origin, MHC class II-restricted antigen presentation, and co-stimulatory capacity. We propose a two-layer model that has not been validated in apCAFs. In this model, the chromatin accessibility of CIITA, CD40, CD80, and CD86 constructs a functional licensing range, while interferon - γ (IFN - γ), interleukin-1 (IL-1), and transforming growth factor - β (TGF - β) guide the formation of specific phenotypes within this range. Insufficient co-stimulation may favor immune tolerance; activation of co-stimulatory programs may support CD4^+^ T-cell help and CD8^+^ T-cell infiltration. Treatment-induced changes in CAF composition may reflect selective survival or genuine state transitions. Distinguishing these mechanisms requires lineage tracing, longitudinal single-cell transcriptomic and chromatin profiling, and patient-derived organoid–fibroblast co-cultures. Neoadjuvant therapy and serial endoscopic sampling provide feasible settings for capturing transient states. This framework supports replacing broad CAF depletion with temporally and state-informed functional reprogramming.

## 1 Introduction

Cancer-associated fibroblasts (CAFs) are a major stromal component of the tumor microenvironment and influence tumor progression and therapeutic resistance through cytokine secretion, extracellular matrix remodeling, and regulation of immune and metabolic niches. Single-cell and functional studies have revealed substantial heterogeneity and plasticity among CAFs, with different subsets either promoting or restraining tumor growth (1–4). This functional diversity may explain the limited efficacy of indiscriminate CAF depletion. The anti-FAP antibody sibrotuzumab showed no meaningful clinical activity in metastatic colorectal cancer (CRC) (5). In pancreatic ductal adenocarcinoma (PDAC) models, depletion of αSMA^+^ myofibroblasts or inhibition of Shh–Smoothened signaling reduced stromal components but increased tumor aggressiveness and shortened survival, indicating that some stromal populations constrain malignant progression (6–9). By contrast, depletion of FAP^+^ stromal cells in Lewis lung carcinoma alleviated immunosuppression and induced tumor necrosis (10). CAF function is therefore determined by cellular state, tissue context, and the surrounding microenvironment.

Single-cell transcriptomic and spatial analyses have identified several recurrent CAF states, including myofibroblastic CAFs (myCAFs), inflammatory CAFs (iCAFs), and antigen-presenting CAFs (apCAFs). These phenotypes are regulated by local signals rather than fixed lineage boundaries. TGF-β suppresses IL-1-driven inflammatory programs and promotes an LRRC15^+^ myCAF-like state (11–15). apCAFs illustrate the difficulty of inferring function from transcriptional identity alone. Although they express major histocompatibility complex class II molecules and possess antigen-presentation-related programs, their immunological effects vary across tumor contexts. MHC class II-positive fibroblasts may support antitumor immunity or promote regulatory T-cell differentiation and immune tolerance.

The apCAF paradox raises questions about whether apCAFs identified across gastrointestinal tumors represent a common cell population or distinct fibroblast states sharing similar antigen-presentation programs. The molecular and microenvironmental factors that determine their immunostimulatory or immunosuppressive activity remain unclear. If apCAFs constitute a reversible, context-dependent state rather than a stable identity, therapies based on limited markers or broad cellular depletion are unlikely to produce consistent outcomes. Resolving this paradox requires a clearer distinction among cellular origin, phenotypic state, and biological function, which may support the development of more precise, context-specific therapeutic strategies.

CAF plasticity has been established. The scope of the suggestions proposed here is narrower: origin, MHC class II restricted presentation, and co stimulatory ability are evaluated as three independent attributes rather than a single label; Read the literature on gastrointestinal apCAF based on the level of evidence provided by each study; And express the obtained framework as a prediction with defined fault conditions.

## 2 The paradox as it currently stands in gastrointestinal cancers

Single-cell RNA sequencing of mouse and human pancreatic ductal adenocarcinoma (PDAC) led Elyada et al. to identify antigen-presenting cancer-associated fibroblasts (apCAFs). These cells express MHC class II molecules and CD74 but lack the canonical co-stimulatory molecules CD40, CD80, and CD86. Functional studies showed that apCAFs activate CD4^+^ T cells in an antigen-dependent manner and promote regulatory T-cell (Treg) differentiation (12, 16–19). In mouse PDAC, a proportion of apCAFs arise from mesothelial cells through an IL-1- and TGF-β-regulated transition. Anti-MSLN treatment inhibited this process, reduced Treg induction, and decreased orthotopic tumor burden (16, 20, 21). Antigen presentation by PDAC-associated apCAFs therefore appears to favor immune tolerance rather than productive antitumor immunity.

Song et al. identified a distinct apCAF population in gastric cancer characterized by high CD74 and HLA class II expression and enrichment of antigen-processing and presentation pathways. Greater apCAF infiltration was associated with earlier-stage disease, longer overall and recurrence-free survival, and improved responses to anti-PD-1-based treatment (22–24). This pattern differs from the tolerogenic activity reported in PDAC. Single-cell studies of gastric and colorectal cancers also demonstrate extensive CAF heterogeneity, yet their classification criteria and functional annotations remain inconsistent (25, 26). In primary liver cancer, treatment can reshape CAF composition, and selected apCAF states have been associated with therapeutic response. Their temporal evolution and functional changes remain poorly defined (27).

The differential effects attributed to apCAF are partly due to inconsistent definitions and partly due to asymmetric evidence. In pancreatic ductal adenocarcinoma, Treg induction has been confirmed in antigen-specific co culture, and anti MSLN therapy reduces Treg induction and *in vivo* tumor burden (12, 16). In cancer, the abundance of apCAF is correlated with T cell infiltration, staging, survival rate, and checkpoint blockade response, without interference or measurement of co stimulatory molecules (22). The correlation between a proven function and a result describes different things, and the differences between them cannot establish biological differences. The ability to co stimulate is a candidate explanation for this difference; The antigen source, local cytokine environment, and resident T cell composition are other factors that have not yet been measured in the gastric population. Marker panels vary across studies, cellular origins are rarely traced, and determinants of antigen-presentation outcomes—including co-stimulatory capacity, antigen source, T-cell interactions, and cytokine context—remain insufficiently examined. MHC class II or CD74 expression alone cannot establish a shared lineage or immune function among apCAFs, nor can it explain their opposing effects across tumor types. **Table 1** summarizes the features evaluated to date and the major remaining evidence gaps.

**Table 1:** The apcaf population reported in gastrointestinal tumors was classified according to the level of evidence.

Tumor type	Defining markers reported	Origin reported	Costimulatory molecules assessed	Functional readout	Association with outcome	Reference
PDAC, mouse and human	MHC class II, CD74, Saa3 low	Not resolved	Yes, classical costimulatory molecules absent	Antigen-specific CD4 T cell activation *in vitro*	Not assessed	(12)
PDAC, mouse and human	MHC class II, MSLN	Mesothelial	Partially	Induction of FOXP3-positive regulatory T cells	Not assessed	(16)
Gastric cancer	CD74, HLA-DRA, HLA-DRB1	Not resolved	Not reported	Association with T cell infiltration	High fraction associated with earlier stage, longer survival, and response to checkpoint blockade	(22)
Primary liver cancer	MHC class II high CAF states	Not resolved	Not reported	Compositional change under therapy	Not assessed	(27)

The difference in reported apcaf function is partly a comparison between the levels of evidence. There is no study to measure the co stimulatory ability and immune results of the same apcaf population.

## 3 A definition problem: apCAF as a state rather than a lineage

### 3.1 What the label currently reports, and what it omits

MHC class II expression alone neither establishes functional antigen-presenting capacity in fibroblasts nor predicts the resulting immune response. Functional assessment should determine whether these cells process antigen or acquire preformed peptide–MHC II complexes, display them to T cells, and provide adequate co-stimulatory signals. Dubrot et al. showed that lymph node stromal cells, including fibroblastic reticular cells (FRCs), acquire peptide–MHC II complexes from dendritic cells while expressing low levels of co-stimulatory molecules. Their interaction with antigen-specific CD4^+^ T cells impaired T-cell survival, proliferation, and recall responses, resulting in antigen-specific tolerance (18, 28–30). Comparable effects have been reported for other nonprofessional MHC II^+^ antigen-presenting cells. Certain epithelial, endothelial, and stromal cells fail to efficiently prime naïve CD4^+^ T cells and can instead promote peripheral tolerance (31). The contrasting effects of apCAFs in pancreatic ductal adenocarcinoma and gastric cancer may therefore reflect differences in co-stimulatory capacity and local accessory signals rather than distinct fibroblast lineages.

These observations support defining apCAFs as a plastic functional state rather than a fixed subtype identified by a limited marker set. This state can be characterized along three axes: cellular origin, MHC class II-restricted antigen-presenting capacity, and co-stimulatory capacity. The central question is not whether apCAFs uniformly promote or suppress antitumor immunity, but how the combination of these properties within a given apCAF population determines its immunological effect.

### 3.2 Technical caveats that inflate the paradox

Variation in apCAF annotation across studies may partly reflect technical limitations of single-cell sequencing. Droplet-based single-cell RNA sequencing is vulnerable to ambient RNA contamination and doublets. Abundant cell-type-specific transcripts may contaminate unrelated droplets, whereas heterotypic doublets generate hybrid expression profiles that distort clustering and cell-type assignment. SoupX and DoubletFinder can reduce these artifacts by correcting ambient RNA signals and detecting doublets, respectively (32, 33). Fibroblasts may also acquire surface MHC class II molecules from professional antigen-presenting cells. MHC II protein detection alone therefore does not establish endogenous expression.

These limitations do not challenge the existence of apCAFs, which have been validated in PDAC by protein-level analyses, antigen-presentation assays, and functional T-cell experiments (12, 16). They do, however, indicate that MHC II-related transcriptional signatures are insufficient for definitive identification. A rigorous framework should integrate evidence of a CIITA-driven endogenous antigen-presentation program, MHC II protein expression, and functional T-cell responses. This approach would distinguish bona fide antigen-presenting fibroblasts from apparent phenotypes caused by ambient RNA, doublets, or intercellular transfer of MHC II molecules.

### 3.3 A permissive layer above the signaling layer

The evidence for epigenetic stability but pharmacological reversibility of fibroblast status comes from non apCAF models. Transient TGF - β 1 stimulation induces LIF and p300 dependent STAT3 acetylation, activates DNMT3B, methylates SHP-1 promoter, and maintains JAK1/STAT3 signaling and pro invasive phenotype; DNMT3B establishes a program, DNMT1 maintains a program, and DNA methyltransferase and JAK combined inhibition can sustainably reverse a program (34–36). In the pancreatic stromal model, activation of vitamin D receptors, all trans retinoic acid, and inhibition of class I HDAC also reversed the established fibroblast state (37, 38). Class I HDAC inhibition attenuates profibrotic and tumor-promoting transcriptional programs, reduces LIF-dependent paracrine signaling, and restrains PDAC progression (39). These studies did not detect MHC class II or co stimulatory sites, nor did they detect apCAFs.

By analogy, we propose a two-layer model for apCAF regulation (**Figure 1**). In apCAF, the chromatin accessibility of CIITA, CD80, CD86, and CD40 has not been analyzed, and there are no reports on apCAF reprogramming experiments. Therefore, each element of the following model is inferred. A permissive epigenetic layer, defined by chromatin accessibility at *CIITA* and co-stimulatory loci such as *CD80*, *CD86*, and *CD40*, determines which functional programs can be engaged. An instructive signaling layer, comprising cues such as IFN-γ, IL-1, and TGF-β, selects specific phenotypes within this accessible range. Stable silencing of co-stimulatory loci could uncouple MHC class II induction from effective helper T-cell activation, limiting the impact of cytokine modulation alone. Accessible but incompletely activated loci may confer greater susceptibility to functional reprogramming. If the model is valid, treatment strategies can be guided by the epigenetic status of apCAFs and prioritize functional remodeling over exhaustion. If the accessibility of these loci proves that there is no distinction between tolerance and immunostimulatory apCAFs, then the model is incorrect.

**Figure 1:**
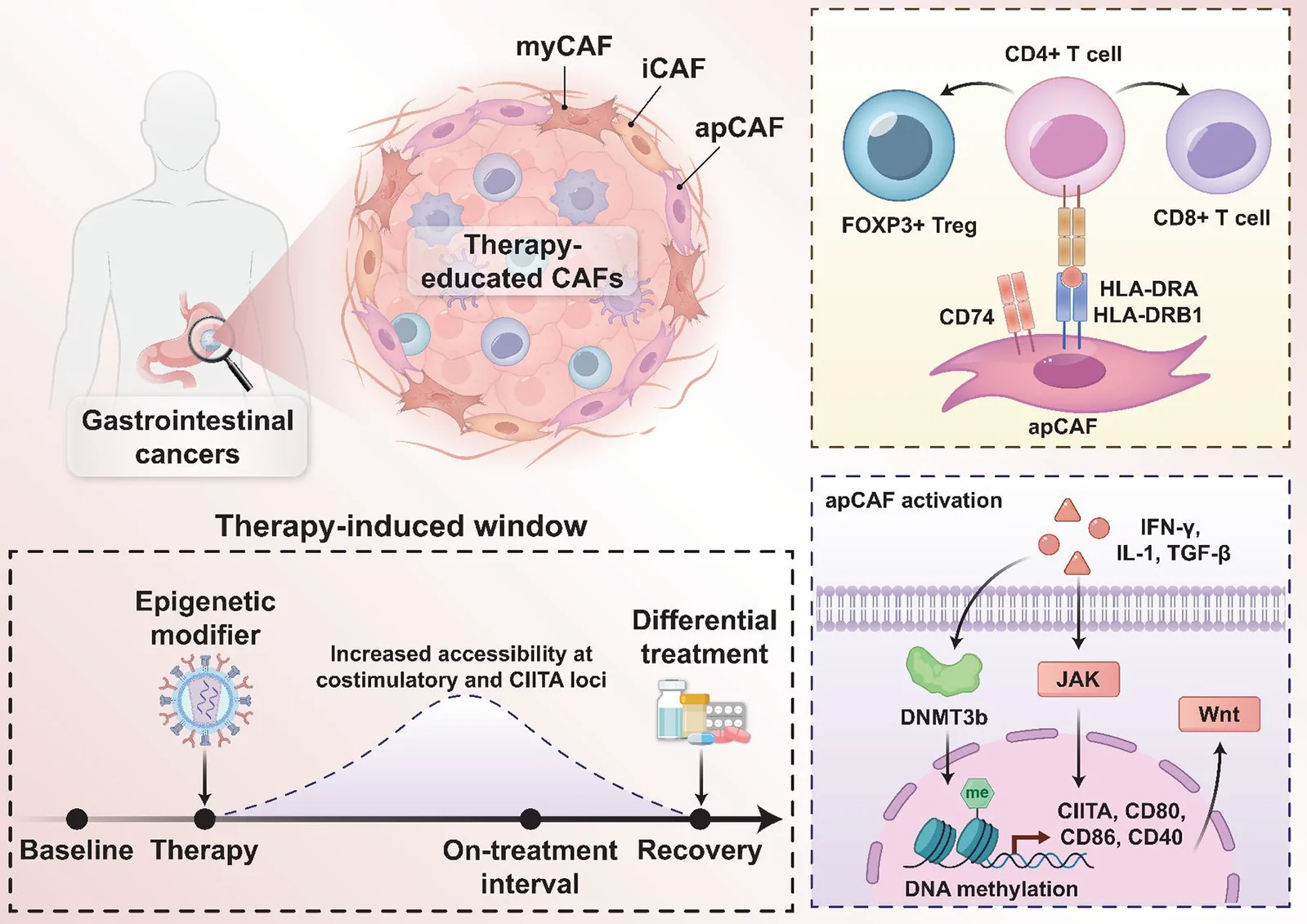
A two-layer model of antigen-presenting cancer-associated fibroblast state. This model is proposed rather than verified. In this note, each element is marked as “observed” (i.e., supported by published experimental data) or “proposed” (i.e., inferred in this opinion article and not verified in apcafs). Observed. Fibroblasts from different sources, including resident tissue fibroblasts, mesothelial cells and stellate cells, tend to be MHC class II molecule positive phenotypes (upper left and center). Apcafs expressing CD74 and HLA-DRA/HLA-DRB1 can present antigen to CD4 T cells. In pancreatic ductal adenocarcinoma, this process can drive Foxp3 positive regulatory differentiation in the absence of classical costimulation (upper right). Treatment can reshape CAF composition (central). In the non apcaf fibroblast model, the DNA methylation dependent phenotypic state can be established through Jak and DNMT3b mediated TGF - β and IL-1 signaling pathways, and this state can be reversed by drug intervention (lower right); This module is for analogy only, and has not been verified in apcafs or MHC class II molecules and costimulatory gene loci. Proposed. It is a hypothesis that the allowed layer is defined as the chromatin accessibility of CIITA and CD80, CD86 and CD40 sites; The accessibility of these loci has not been described in apcafs, and the tolerance and immunostimulatory apcaf populations have not been compared. In the licensed configuration, accessible and participating costimulatory sites translate the same class II expression into CD4 help and CD8 infiltration (upper right corner, CD8 branch), which is predicted, but has not been confirmed. The window of treatment induction (lower left corner) includes the transient increase in accessibility after cytotoxic, matrix directed or checkpoint directed treatment, and the predictive benefits of combined epigenetic and differentiation directed interventions within this time interval. The accessibility curve displayed is a schematic diagram and does not represent the measured data.

## 4 Selection or conversion? What the plasticity data can and cannot show

Therapy can markedly reshape CAF composition, but direct interconversion between CAF states remains unproven. TGF-β blockade reduces myCAFs and is accompanied by the emergence of interferon-licensed CAFs (ilCAFs), increased CD8^+^ T-cell infiltration and activation, and reduced spatial and functional immune exclusion (40). Similar remodeling occurs in high-grade serous ovarian cancer. Platinum- and taxane-based chemotherapy decreases ANTXR1^+^ ECM-myCAFs and relatively enriches ANTXR1^-^ iCAFs. Residual ECM-myCAFs continue to suppress CD8^+^ T-cell cytotoxicity through a YAP1-dependent mechanism (41). Treatment-associated changes in CAF composition have also been reported in primary liver cancer (27).

These compositional shifts may reflect selective survival of pre-existing states or phenotypic conversion of individual CAFs. Both mechanisms produce similar patterns in cross-sectional single-cell datasets but have different therapeutic implications. Selective survival would favor strategies that eliminate detrimental populations while preserving beneficial ones. State conversion would support interventions designed to reprogram CAF function.

Distinguishing these mechanisms requires longitudinal fate-mapping evidence. Fibroblast-specific lineage tracing and pre-treatment clonal barcoding can directly track cells and their progeny. Paired pre- and post-treatment single-cell transcriptomic or ATAC-sequencing data, combined with trajectory analysis, can reveal state continuity and regulatory changes but cannot independently establish cellular ancestry. Until such approaches are applied directly to apCAFs, their therapeutic reprogramming should be considered a testable hypothesis supported by indirect evidence rather than a demonstrated cell-fate transition.

## 5 From depletion to licensing: a framework and its predictions

The framework we propose can be stated compactly. Cytotoxic chemotherapy, TGF-beta-directed agents, and checkpoint blockade transiently remodel the fibroblast regulatory landscape, and during that interval the permissive layer described above is more accessible than at baseline. In this interval, the elimination of epigenetic modifiers of inhibitory markers on costimulatory and CIITA related sites and the binding of directional differentiation signals with executive layer are expected to transform apcaf from tolerant configuration to licensed configuration without exhausting them. The prediction assumes that these markers exist at these sites of apcafs, and treatment will temporarily alleviate them. Neither is shown. The clinical objective is not fewer fibroblasts but fibroblasts that finish the conversation they start.

Three predictions follow, and each can fail.

1. Immune-activating and tolerogenic apCAF populations should differ more in chromatin accessibility at CD80, CD86, CD40, and CIITA than in the level of MHC class II expression itself. If accessibility at these loci is indistinguishable between gastric and pancreatic apCAFs, the model is wrong.
2. Accessibility at these loci should increase transiently after cytotoxic or stroma-directed therapy, and should do so before subtype proportions restabilize. If accessibility tracks proportion with no lead interval, there is no window to exploit.
3. Pairing an epigenetic modifier with a differentiation cue during that interval should increase antigen-specific CD4 help and CD8 infiltration more than either intervention alone and more than the same combination given at baseline. If timing is irrelevant, the window concept adds nothing.

Two risks deserve to be stated rather than deferred. Broad epigenetic agents act on tumor cells and lymphocytes as well as fibroblasts, so any observed benefit requires fibroblast-restricted genetic controls before it can be attributed to stromal reprogramming. And enhancing class II presentation by a stromal cell of self-derived antigen carries a plausible autoimmune cost, particularly in the gastrointestinal tract, where tolerance to luminal antigen is actively maintained.

## 6 Why gastrointestinal cancers are the appropriate testbed

Evidence for therapy-induced apCAF reprogramming requires longitudinal detection of transient cellular states. Neoadjuvant treatment of gastrointestinal tumors provides a practical framework for such studies. In esophageal cancer, serial biopsies obtained before and during treatment, together with surgical specimens, have been used to track microenvironmental changes. Gastric cancer studies have performed paired multi-omic profiling before and after neoadjuvant chemotherapy or immunochemotherapy. Repeated endoscopic sampling enables similar longitudinal analyses in rectal cancer (42–46). These predefined sampling schedules permit comparisons among pretreatment, on-treatment, and surgical specimens, facilitating the detection of transient CAF states and therapy-induced microenvironmental remodeling. Patient-derived tumor organoids co-cultured with matched fibroblasts have also been established in pancreatic and colorectal cancers to study drug responses and stroma-mediated resistance. These models provide patient-matched *ex vivo* platforms for testing candidate interventions (47–49).

Noninvasive monitoring is currently unable to define the apcaf status. FAP targeted pet reflects the overall activity of the matrix, while apcaf is heterogeneous in FAP + compartment and FAP is usually low, so the intake is at best a guide to the sampling time point (50–52). No circulating apcaf biomarkers were verified. Therefore, continuous tissue sampling is still the only information method, which is why gastrointestinal tumors are an easy to handle setting.

## 7 Discussion

The divergent functions attributed to apCAFs may partly reflect limitations in their definition. Classifying apCAFs as either promoters or suppressors of antitumor immunity assumes that cells identified across studies represent the same biological entity, an assumption not yet supported in gastrointestinal tumors. MHC class II expression and related transcriptional signatures identify fibroblasts with an antigen-presentation-associated phenotype but do not establish cellular origin, functional antigen-presenting capacity, or immune outcome. The consequences of antigen presentation also depend on co-stimulatory activity and the local microenvironment. Studies of other CAF states suggest that epigenetic programs may constrain responses to external signals, but this mechanism remains unverified in apCAFs.

apCAF classification should move beyond restricted marker sets toward multidimensional functional characterization. Relevant criteria include cellular origin, CIITA-centered endogenous MHC class II programs, surface MHC class II expression, co-stimulatory status, and functional T-cell responses. Antigen-presenting and co-stimulatory capacities should be assessed independently. This framework would clarify whether apCAFs described across studies are biologically comparable and may reconcile the tolerogenic phenotype reported in PDAC with their association with favorable antitumor immunity in gastric cancer, without assuming a shared lineage or function.

This framework also changes the rationale for CAF-directed therapy. Broad stromal depletion has produced inconsistent benefits and, in some settings, increased tumor aggressiveness. Selective targeting of tumor-promoting CAF states may remain valuable. Functional reprogramming offers another possibility: preserving CAFs while shifting tolerogenic antigen-presenting states toward programs that support effector T-cell responses. General CAF populations exhibit epigenetic stability and pharmacological plasticity, yet it remains unclear whether treatment-associated compositional changes reflect selective survival or genuine state conversion. Direct evidence that apCAFs can be reprogrammed is also lacking. Neoadjuvant treatment, serial tissue sampling, and patient-derived organoid–fibroblast co-culture systems provide practical platforms for testing this hypothesis in gastrointestinal tumors. Here are three limitations. Caf plasticity is established, not new. What is added here is a separate attribute and a set of refutable predictions. The two-layer model is deduced from the non apcaf fibroblast model, which is not directly supported in apcaf. The findings of pancreas and stomach have different degrees of evidence, so the ability of CO stimulation is still a candidate explanation, rather than a conclusion. The central priority is not simply to identify additional markers, but to define how apCAF states arise, whether they interconvert, and whether such transitions can be safely exploited to enhance antitumor immunity.

## Data Availability

The original contributions presented in the study are included in the article/supplementary material. Further inquiries can be directed to the corresponding author.
